# Targeting myeloid cells in pancreatic ductal adenocarcinoma: from primary tumors to liver metastasis

**DOI:** 10.3389/fimmu.2025.1555036

**Published:** 2025-05-16

**Authors:** Ruining Gong, Ying Chen, Chang Li, Huan Zhang, Zimin Liu, Qian Yu

**Affiliations:** ^1^ Gastrointestinal Cancer Institute (Pancreatic Disease Institute), the Affiliated Hospital of Qingdao University, Qingdao, China; ^2^ Department of Endocrinology and Metabolism, the Affiliated Hospital of Qingdao University, Qingdao, China; ^3^ School of Biomedical Sciences, the Chinese University of Hong Kong, Hong Kong, Hong Kong SAR, China; ^4^ Department of Food Science and Nutrition and Research Center for Chinese Medicine Innovation, the Hong Kong Polytechnic University, Hong Kong, Hong Kong SAR, China; ^5^ Tumor Immunology and Cytotherapy of Medical Research Center, Shandong Provincial Key Laboratory of Clinical Research for Pancreatic Diseases, the Affiliated Hospital of Qingdao University, Qingdao, China

**Keywords:** pancreatic cancer, immunotherapy, macrophage, monocyte, liver metastases

## Abstract

Pancreatic ductal adenocarcinoma (PDAC) remains one of the malignancies with the highest mortality rates, and outcomes are particularly poor in cases of liver metastasis. Early or recurrent metastatic PDAC significantly worsens patient outcomes and presents substantial clinical challenges. Checkpoint-based immunotherapy has largely been ineffective for most pancreatic cancer patients. This ineffectiveness is not well understood, as clinical trials often involve patients with advanced diseases, such as liver and peritoneal metastases, while most preclinical studies focus on primary tumors. Recent findings indicate that the immunosuppressive tumor microenvironment (TME) is a major obstacle to effective immunotherapy in PDAC, with the metastatic immune microenvironment differing significantly from that of primary tumors. This review explores the distinct immunosuppressive mechanisms at various stages of PDAC progression, including primary tumors, pre-metastatic niches, and metastatic sites. Myeloid cells, such as tumor-associated macrophages (TAMs) and myeloid-derived suppressor cells (MDSCs), play pivotal roles in shaping the TME and suppressing anti-tumor immunity. Particular focus is placed on current clinical immunotherapy strategies targeting myeloid cells, and combinations with conventional chemoradiotherapy, considering contemporary knowledge and future trends. Advancements in single-cell RNA sequencing (scRNA-seq) and spatial transcriptomics have provided deeper insights into the molecular intricacies and diversity of PDAC, revealing potential therapeutic targets. Innovative strategies targeting myeloid cells, including CD40 agonists and CSF-1R inhibitors, are being explored to reprogram the TME and enhance the efficacy of immunotherapies.

## Introduction

Pancreatic adenocarcinoma (PDAC) presents significant treatment challenges due to its prolonged asymptomatic phase, early liver metastasis, and high resistance to therapies (1). Post-surgical survival rates are low, with only 34% of patients surviving one year after a liver metastasis diagnosis (2). Chemotherapy and surgery continue to be the primary treatment options; however, only 15%–20% of patients qualify for surgical intervention at the time of diagnosis (3). Although immunotherapy has shown efficacy in treating other malignancies such as metastatic melanoma, renal cell carcinoma, and non-small cell lung cancer (4, 5), its application in PDAC has yielded disappointing results (6–9). A 2020 study found that only 1.01% (636 out of 63,154) of post-surgical PDAC patients received immunotherapy (9). Combined with chemoradiation, it improved median overall survival (29.31 vs. 23.66 months, p < 0.0001) and survival rates at 1 year (88% vs. 81%) and 2 years (60% vs. 49%) (9). However, trials like a 2019 durvalumab study and a 2010 ipilimumab trial showed no survival benefit in metastatic pancreatic cancer (7, 8). This ineffectiveness may be attributed to the advanced disease stages of patients in clinical trials, which often include liver and peritoneal metastases, while most preclinical studies focus on primary tumors. Moreover, preclinical studies suggest that combining immune checkpoint inhibitors with myeloid cell targeting could offer more promising treatment outcomes (10, 11).

The establishment of liver metastatic tumors requires cancer cells to navigate complex steps within a foreign microenvironment, evade immune detection, and successfully colonize distant tissues. The process of liver metastasis is influenced by both tumor-intrinsic and extrinsic factors, beyond mere genomic alterations. The tumor microenvironment (TME) is critical for cancer initiation and progression (12–14). PDAC exhibits low immunogenicity, which hinders the production and release of new antigens, leading to reduced levels of tumor-infiltrating lymphocytes (TILs) (15, 16). Cytotoxic T lymphocytes are essential anticancer effector cells, and their activation by antigen-presenting cells (APCs) is crucial for effective T cell responses (17). The coexistence of inflammatory and immunomodulatory signals in the pancreatic TME leads to dysregulated repair and cytotoxic responses (18). Within the TME, tumor-associated macrophages (TAMs) are the most abundant antigen-presenting cells (APCs), yet they often have poor antigen-presenting capacity, acting as the main immune infiltrate and being associated to poor patient outcomes (19, 20). Embryo-derived tissue-resident macrophages (21–23), and bone-marrow-derived precursors, such as circulating Ly6C^+^ monocytes, may sustain TAM levels (24–26). Studies suggest that macrophages of distinct ontogenic origins exhibit divergent functional roles, depending on the tumor type. Macrophages in tumors and at (pre)metastatic sites possess dual origins and distinct functions. Effective therapeutic intervention therefore requires a thorough understanding of the sources that sustain macrophages in malignant tissues, and elucidating the heterogeneity of TAM origin and function at each metastatic step could provide valuable insights for PDAC treatment.

This review delves into the characteristic features of immunosuppression at various stages of PDAC progression, including primary tumors, pre-metastatic niches, and metastatic sites. Myeloid cells, such as tumor-associated macrophages (TAMs) and myeloid-derived suppressor cells (MDSCs), play pivotal roles in shaping the TME and restraining anti-tumor immunity. Recent advancements in single-cell RNA sequencing (scRNA-seq) and spatial resolved transcriptomic analysis have provided deeper insights into the molecular intricacies and diversity of PDAC, revealing potential therapeutic targets. Innovative strategies targeting myeloid cells, including CD40 agonists and CSF-1R inhibitors, are being explored to reprogram the TME and enhance the efficacy of immunotherapies.

## Stage-specific immunosuppressive landscapes in PDAC progression

**Figure 1:**
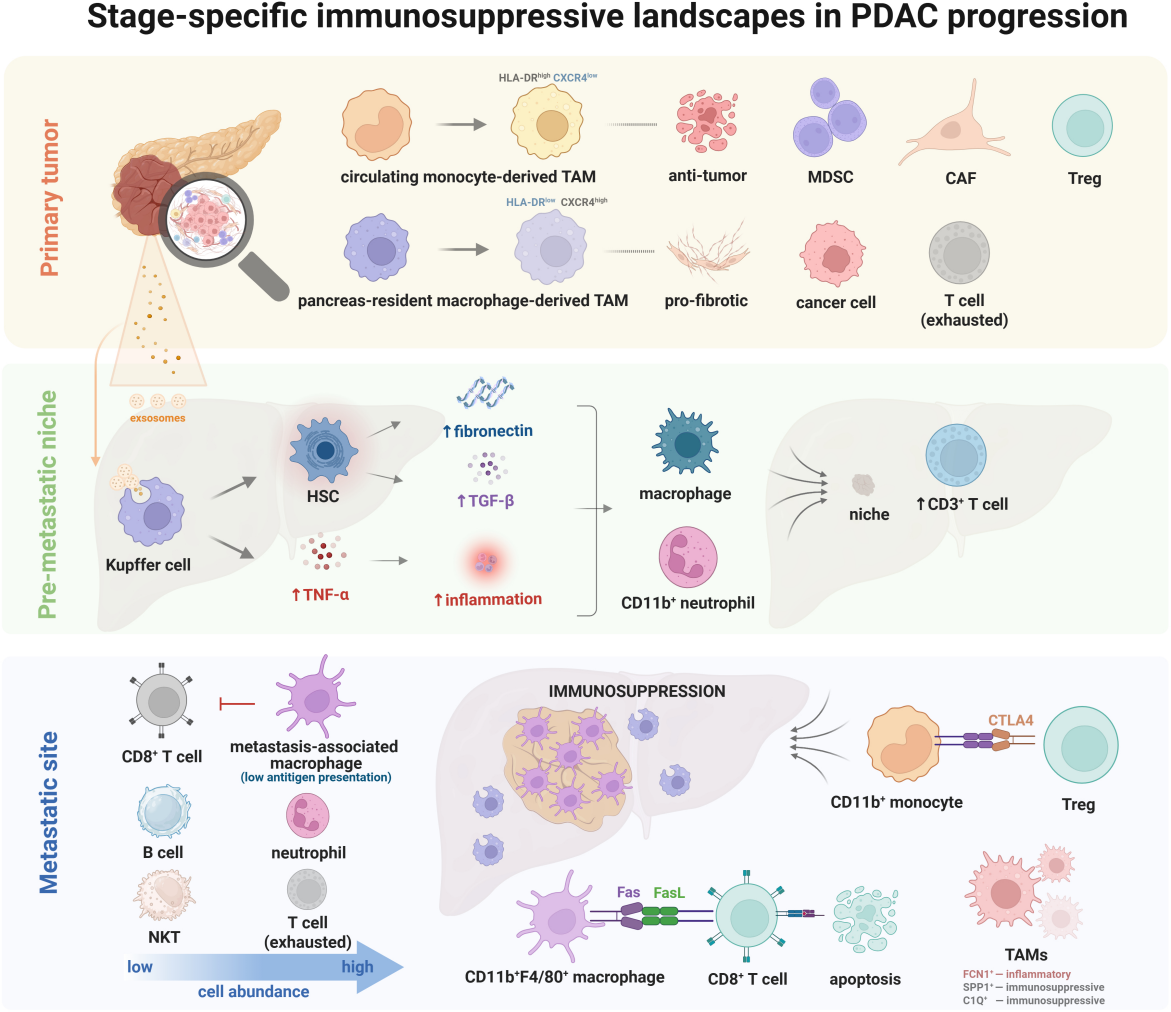
Schematic representations of the TME at three critical stages: primary tumor, pre-metastatic niche, and metastatic site.

To provide a comprehensive visual summary of the immunosuppressive landscape in PDAC progression, we propose schematic representations of the TME at three critical stages: primary tumor, pre-metastatic niche, and metastatic site (**Figure 1** **).**

### Primary tumor microenvironment

The PDAC primary tumor exhibits a unique tripartite immunosuppressive architecture dominated by myeloid cells, such as TAMs, MDSCs, and neutrophils, dominate the immune landscape in pancreatic tumors (27, 28). This has led to extensive research into their roles in PDAC biology, particularly their impact on immune suppression. In PDAC cells, oncogenic KRAS signaling induces the secretion of GM-CSF, which attracts immunosuppressive MDSCs (28, 29). TAMs, originating from both tissue-resident macrophages and circulating monocytes, play essential yet potentially distinct roles in shaping the TME and inhibiting the adaptive immune response (27). In an orthotopic mouse model of PDAC, monocyte-derived TAMs (HLA-DR^high^CXCR4^low^) exhibited an antitumor phenotype, characterized by strong antigen-presenting capabilities (27). In contrast, pancreas-resident macrophages (HLA-DR^low^CXCR4^high^) developed a pro-fibrotic transcriptional profile, thereby promoting a desmoplastic reaction (27). However, these findings need validation in human PDAC tumors.

The limited success of immune checkpoint blockade in PDAC is complex, involving deficiencies in natural T cell responses, partly due to ineffective T cell priming and/or T cell exclusion (30, 31). New strategies targeting MDSCs and TAMs to induce anti-tumor immunity and enhance checkpoint inhibition in pancreatic cancer are being explored (11, 28, 29, 32–34). For instance, arginase 1 in TAMs suppresses immune responses by inhibiting T cell activation, and systemic treatment with the arginase inhibitor CB-1158 sensitizes PDAC to anti-PD1 immune checkpoint blockade (35). Additionally, novel agonists targeting the CD11b/CD18 receptor on tumor-infiltrating myeloid subsets have demonstrated promising preclinical results and are currently undergoing clinical trials (10, 36). Another strategy involves using CD40 agonists to reprogram macrophages into a less immunosuppressive state, which facilitates CD8+ T cell infiltration and transforms a ‘cold’ tumor into a ‘hot’ tumor (37). Thus, effective cancer immune surveillance in pancreatic tumor resistant to checkpoint inhibition can be achieved by coactivating complementary myeloid signaling pathways.

Advancements in spatial resolved transcriptomics and scRNA-seq have revealed the molecular complexity (**Table 1**), tumor heterogeneity, and functional specificity of resected PDAC, showing dynamic changes in the TME as the disease progresses (38–42). Typically, ductal cells are not predominant; instead, there is an accumulation of tumor-infiltrating immune cells, cancer-associated fibroblasts (CAFs), and dense extracellular matrices from early to late PDAC stages. Anti-tumor immune responses arise but are suppressed by regulatory T cells (Tregs), exhausted T cells, and TAMs as the tumor progresses (38, 43, 44). Moffitt et al. combined 92 scRNA-seq samples from seven studies to develop a reproducible PDAC atlas, emphasizing tumor-TME interdependence (45). This atlas offers a detailed overview of the signaling interactions that regulate PDAC. It was observed that patients with activated stroma exhibit increased M2-like macrophages and Tregs. In contrast, patients with ‘normal’ stroma show M1-like macrophage recruitment and elevated levels of effector and exhausted T cells (39). By integrating clinical histopathology with multi-tiered OMICs analyses, Barbara et al. identified ‘subTMEs,’ with regional relationships to tumor immunity, subtypes, differentiation, and treatment response. The study demonstrated that multiple ecotypes within individual patients provide a more intricate perspective than the mutually exclusive subtypes identified by scRNA-seq studies, thereby highlighting the molecular intricacies and heterogeneity of the TME (46). This complexity can only be fully understood through spatial analysis (47). Collectively, these studies underscore the necessity of spatial sequencing to characterize the polyclonal and heterogeneous nature of PDAC tumors.

**Table 1:** Advantages and disadvantages of scRNA-seq and spatial transcriptomics (46).

Parameter	scRNA-seq	Spatial transcriptomics
Spatial Context	Lost during dissociation.	Retained *in situ*.
Resolution	Single-cell.	Varies (subcellular to multi-cell).
Applications	Cell identity, heterogeneity, dynamic changes.	Tissue organization, spatial niches.
Scalability	Highly scalable, can profile hundreds of thousands of cells efficiently.	Limited by method: array-based SRT like Visium can cover large tissue areas, but imaging-based methods may be slower and less scalable.
Complexity	Established protocols.	Higher technical challenges.
Platform	E.g., 10x Genomics Chromium Controller, established and widely used.	E.g., Visium (10x Genomics, 55 μm), High-resolution Visium (10x Genomics, 2 μm), GeoMx (Nanostring), CosMx, MERSCOPE, still evolving.
Challenges	Requires tissue dissociation, may induce stress/cell death, not suitable for neurons without specialized protocols; high dropout rates.	Trade-offs between resolution and gene coverage; imaging-based methods require long microscopy times (hours to days); array-based methods may mix mRNA from multiple cells, risking mRNA diffusion.

### Pre-metastatic niche

Only 25% of PDAC patients are diagnosed at a stage amenable to complete surgical resection, with the majority of research focusing on early-stage disease, which represents a small portion of the patient population (45, 48–50). Understanding how PDAC cells evade immune attacks during circulation is a critical future challenge. After escaping the primary tumor, cancer cells must avoid immune detection and elimination to reach and colonize distant organs (51–53). Recent studies suggest that metastatic sites, especially the liver, undergo microenvironmental changes that support metastatic growth even before metastasis is clinically evident. These changes, known as “pre-metastatic niches,” are driven by signals from the primary pancreatic tumor, which have tumor-supportive and immune-suppressive characteristics (54–58). These signals reprogram resident cell phenotypes, alter the extracellular matrix, and affect immune cell infiltration, thereby supporting the expansion of disseminated tumor cells (51, 59, 60).

Immunosurveillance can impede each phase of tumor progression, including metastasis. Consequently, tumors and their metastatic counterparts must devise mechanisms to evade immune destruction, such as creating an immunosuppressive pre-metastatic niche. Regulatory or immunosuppressive cells, such as MDSCs, macrophages, and Treg cells within this niche, can inhibit anti-tumor immune responses (61, 62). In particular, macrophage recruitment to the liver is a key feature of the early liver pre-metastatic niche, evident even during the PanIN stages (54), as demonstrated by Bruno et al. for the first time that the uptake of PDAC-derived exosomes by Kupffer cells leads to the secretion of transforming growth factor and increased fibronectin production by hepatic stellate cells. This process attracts bone marrow-derived macrophages (and potentially neutrophils) to the liver, creating a favorable environment for liver metastasis. This mechanism was further corroborated by a recent study demonstrating that fatty acid cargo in tumor-derived extracellular vesicles and particles, particularly palmitic acid, induces TNFα secretion by Kupffer cells, generating a pro-inflammatory microenvironment (63). More recently, it was revealed that pre-metastatic livers in PDAC patients show a significant inflammatory response, marked by increased infiltration of CD11b^+^ neutrophils and macrophages, as well as CD3^+^ lymphocyte subsets at the time of resection, compared to non-cancerous livers (64). This was observed regardless of whether the future metastatic sites were the lungs or liver (64). This study highlights that myeloid cell infiltration in the liver is an early indicator of PDAC metastasis and a defining feature of the liver pre-metastatic niche. This event precedes T cell infiltration, which occurs later in the progression of pancreatic cancer and may signal anti-tumor activity. Nevertheless, the early stages of metastasis are marked by increased neutrophil infiltration and neutrophil extracellular traps (NETs) formation, although the specific role of NETs in liver metastasis remains to be clarified (64).

### Metastatic liver microenvironment

Since radical surgery is not typically used for advanced or metastatic stages, it is rather difficult to obtain matched primary and metastatic samples for spatial transcriptomics. Bulk RNA sequencing studies have revealed that metastatic TMEs exhibit pronounced immunosuppression, rich in metastasis-associated macrophages (MAMs) and neutrophils, and have low levels of T cells, B cells, and natural killer cells (65, 66). However, bulk transcriptomics average gene expression, missing distinct microenvironmental ecosystems. To thoroughly comprehend the exact spatial and cellular contexts of these molecular alterations, it is vital to address localized heterogeneous signals (67). ScRNA-seq has revealed the diversity within PDAC (39, 68–72). Although some investigations have concentrated on primary pancreatic tumors, the spatial distribution of different cell clusters and the biology of metastasis have only recently been disclosed (46). In metastatic PDAC livers, macrophage populations with opposing roles can coexist in the same tumor (46, 66, 73). Specifically, Kupffer cells, found at the periphery of metastatic lesions, display inflammatory and pro-fibrotic signatures while monocyte-derived macrophages (MoMs), located in the core areas, show diverse immunostimulatory and immunosuppressive signatures (66, 73). Early in PDAC metastasis, a subpopulation of MoMs with low antigen presentation gene expression and an efferocytosis gene signature compromised CD8^+^ T cell activation thus leading to immune suppression (66). Khaliq et al. employed spatially resolved transcriptomics and ecotype assessments to assess the metastatic TME, identifying a unique ecotype at the invasive tumor margin with a variety of immune-related genes (46). This ecotype comprises both tumor-promoting factors, such as M2-like TAM and Treg cells, and tumor-suppressing components, including cytotoxic T cells and M1-like TAM, which cannot be entirely represented by single-cell RNA sequencing alone, underscore this ecotype as a promising target for immunotherapy. In addition, TAM subtypes with differing functions, such as inflammatory FCN1^+^ and immunosuppressive SPP1^+^ and C1Q^+^ phenotypes, co-occur within tumors. This further supports findings that TAMs with both roles exist simultaneously in the same TME. Taken together, spatially resolved transcriptomics of matched primary and metastatic PDAC samples offer critical insights into metastatic PDAC biology and are crucial for future therapeutic research. However, the current 55 μm spatial resolution might not sufficiently represent the intricacies of cellular interactions, underscoring the necessity for higher-resolution spatial transcriptomics. Future research should also explore the roles of Kupffer cell subpopulations in PDAC liver metastasis and develop therapies targeting immunosuppressive macrophages or their signaling pathways that enable these immunosuppressive traits. Comprehending the spatial organization of tumor ecosystems and the dynamics of cellular interactions is essential for deepening our knowledge of metastatic PDAC biology and the development of effective therapies.

Targeting the immunosuppressive environment has shown promise as a therapeutic approach for various cancers (74, 75). However, in primary PDAC, the effectiveness of immunotherapies is limited due to the desmoplastic and nonimmunogenic tumor microenvironment, which lacks CTL infiltration (76–79). Although it has been proposed that liver metastasis correlates with a poor response to immunotherapy in cancer patients (80), recent studies have found that the metastatic TME contains a relatively higher infiltration of immune cells, including exhausted tumoricidal CTLs, compared to primary PDAC (81), suggesting that targeting the immunosuppressive microenvironment could be a more feasible and viable strategy for treating liver metastasis of PDAC (52, 82). Paradoxically, while liver metastases exhibit localized immune infiltration, they concurrently induce systemic immunosuppression, leading to a widespread reduction in T cells and decreased effectiveness of immunotherapy in preclinical models (80, 83). Lee et al. were the first to report that immune suppression was antigen-specific and involved the coordinated activation of Tregs along with the modulation of intratumoral CD11b^+^ monocytes (83). Yu et al. further identified hepatic monocyte-derived CD11b^+^F4/80^+^ macrophages as key mediators that trigger antigen-specific CD8^+^ T cell apoptosis through the Fas–FasL pathway within the liver metastatic microenvironment. However, these findings require further investigation and validation in human metastatic PDAC contexts (80).

In PDAC liver metastasis, monocytes and macrophages accumulate and often exhibit an immunosuppressive phenotype, promoting tumor growth and reducing immunotherapy effectiveness (84–87). However, when activated appropriately, macrophages can mediate the phagocytosis of cancer cells, engage in cytotoxic tumor killing, and interact effectively with components of both the innate and adaptive immune systems (87). Genetic engineering to induce cytokine expression in liver macrophages, such as Kupffer cells and TAMs, could transform the liver tumor TME into an immune-reactive state, enhancing protective immune responses to tumor (88). Astuti et al. recently demonstrated that efferocytosis reprograms macrophages towards an immunosuppressive phenotype. Inhibiting efferocytic macrophages, either genetically or pharmacologically, has been shown to restore tumor immunity in early PDAC liver metastasis and hinder metastatic growth (66). A recent study pinpointed macrophage-derived granulin as a key factor in T-cell exclusion in metastatic PDAC. It also highlighted the potential of targeting granulin alongside the immune checkpoint inhibitor αPD-1 to treat metastatic PDAC (81). A novel approach involving liver macrophage-targeted IFNa gene transfer was devised to modify KCs adjacent to liver metastases as well as TAMs in preclinical metastasis mouse models. Although some mice showed resistance due to impaired MHC-II-restricted antigen presentation in APCs, combining CTLA-4 blockade with IFNα lentivirus overcame this resistance, activating both innate and adaptive immune responses (89). These findings support developing tailored approaches that focus on suppressive macrophage clusters or the factors that convert macrophages into immune-suppressing cells in metastatic PDAC settings.

## Clinical strategies and their mechanistic insights

The following table summarizes the latest clinical strategies targeting myeloid cells in PDAC, detailing their targets, mechanisms, and current trial status (**Table 2**). It includes CD40 agonists, CSF-1R inhibitors, CD11b agonists, CCR2 inhibitors, BTK inhibitors, and emerging chemokine receptor inhibitors, such as CXCR2 and CXCR4 antagonists, highlighting the diverse immune modulating approaches to reprogram the immunosuppressive tumor microenvironment.

**Table 2:** Clinical strategies targeting myeloid cells in PDAC.

Strategy	Target	Mechanism	Status	NCT number
CD40 agonists (e.g., mitazalimab)	CD40 on macrophages and other immune cells	Activates macrophages to M1 phenotype, enhances T cell priming (90, 91)	In phase Ib/II trials	NCT04888312
CSF-1R inhibitors (e.g., IMC-CS4)	CSF-1R on macrophages	Depletes TAMs, reprograms remaining macrophages to be anti-tumor (32)	Phase I trial (complete)	NCT01346358
CD11b agonists (e.g., GB1275)	CD11b on myeloid cells	Repolarizes TAMs, enhances T cell immunity (10)	Phase I trial (complete)	NCT04060342
CCR2 inhibitors (e.g., PF-04136309, BMS-813160)	CCR2 on monocytes	Blocks monocyte recruitment to the tumor (92)	phase Ib/II (complete), with some toxicity concerns	NCT02732938 NCT03496662
BTK inhibitors (e.g., ibrutinib)	Bruton’s tyrosine kinase in immune cells	Modulates myeloid and T cells to enhance anti-tumor immune responses (93)	In phase III trials	NCT02436668
CXCR2 inhibitors (e.g., AZD5069)	CXCR2 on neutrophils and MDSCs	Inhibits neutrophil and MDSC recruitment to the TME (94), enhances T cell infiltration and chemotherapy/immunotherapy efficacy (95)	phase Ib/II (complete)	NCT02583477
CXCR4 inhibitors (e.g., motixafortide)	CXCR4 on immune and tumor cells	Disrupts CXCR4–CXCL12 axis, inhibits tumor cell homing, metastasis, and immunosuppression, enhances anti-PD-1/PD-L1 responses (96)	phase IIa (complete)	NCT02826486

### CD40 agonists

Emerging strategies to reprogram TAMs through phagocytic activation have opened new frontiers in PDAC immunotherapy (97). Pioneering studies in pancreatic cancer patients revealed that anti-CD40 agonistic antibodies not only stimulate macrophage-mediated tumor cell engulfment but also enhance dendritic cell (DC) cross-presentation and T-cell priming (90). While it was first assumed these antibodies exclusively targeted macrophages, subsequent investigations demonstrated they also enhance dendritic cell (DC) function and improve T-cell priming (98, 99). CD40 agonists, such as mitazalimab, target the CD40 receptor on macrophages and other immune cells, transforming TAMs from an M2 (immunosuppressive) to an M1 (immune-supportive) phenotype. This shift facilitates the degradation of fibrotic stroma, enhances tumor sensitivity to chemotherapy, and improves T cell infiltration (90, 91). Driven by preclinical observations (98–100), the first-in-human dose-finding study of APX005M (agonistic CD40 monoclonal antibody) has shown on-target effects and a manageable safety profile (101). The combination of CD40 agonists with gemcitabine-based chemotherapy has shown promise in patients with metastatic PDAC (102, 103). However, the structural variations among CD40 agonists impact their safety profiles, with systemic administration potentially leading to dose-limiting immune-related adverse effects (37). Mitazalimab, a second-generation CD40 antibody, has demonstrated clinical activity and a favorable safety profile in advanced-stage solid tumors (101, 104). Supported by preclinical data (105), a phase II trial combining mitazalimab with modified FOLFIRINOX in metastatic PDAC patients showed manageable safety and anti-tumor activity, supporting further development in a phase III trial (106).

### CSF-1R inhibitors

CSF-1R inhibitors, such as IMC-CS4, target the colony-stimulating factor 1 receptor on macrophages, depleting protumor TAM subsets and reprogramming remaining macrophages to bolster tumor-suppressing immunity. This enhances interferon responses and increases cytotoxic T lymphocyte (CTL) infiltration, preventing tumor growth (32). A recent trial combining IMC-CS4 with GVAX, cyclophosphamide, and pembrolizumab in PDAC patients showed increased CTL levels, suggesting macrophage reprogramming rather than depletion. This suggests that IMC-CS4 reprogrammed macrophages rather than depleting them (107). However, its effectiveness in metastatic PDAC remains uncertain, indicating a need for further validation.

### CD11b agonists

CD11b agonists, such as GB1275 and ADH-503, target the CD11b receptor on myeloid cells, repolarizing TAMs to enhance antitumor T cell immunity. This strategy makes checkpoint inhibitors effective in previously unresponsive PDAC models by reprogramming immunosuppressive myeloid cells (10). GB1275 is in early clinical development, with preclinical data supporting its potential in immunotherapeutic combinations (36). This approach is an unexpected detail, as it highlights a novel target for enhancing PDAC immunotherapy, though its clinical impact is still being explored.

### CCR2 inhibitors

Despite limited clinical benefit and some off-target toxicities in early trials, CCR2 inhibitor therapy remains a promising approach for PDAC due to its ability to disrupt the CCL2–CCR2 chemokine axis, which recruits immunosuppressive TAMs to the TME. In a phase Ib trial (NCT01413022), the CCR2 inhibitor PF-04136309 combined with FOLFIRINOX in patients with borderline resectable or locally advanced PDAC achieved a 49% objective response rate and a 97% disease control rate, with manageable toxicities, demonstrating reduced TAM infiltration and increased effector T cell presence (108). A phase Ib trial (NCT02732938) evaluating PF-04136309 with nab-paclitaxel and gemcitabine in metastatic PDAC reported improved overall survival (29% vs. 19% at 12 months) compared to historical chemotherapy controls, though pulmonary toxicity was noted (109). Preclinical studies further support CCR2 inhibition’s potential to enhance anti-PD-1 immunotherapy and chemotherapy by increasing CD8+ T cell recruitment and reducing regulatory T cells or TAMs (110, 111). These findings, coupled with a recently completed trial like NCT03496662 (BMS-813160 with nivolumab and chemotherapy), suggest that optimizing CCR2 inhibitor dosing and combination strategies could overcome current limitations, making this approach a promising avenue for further investigation.

### BTK inhibitors

BTK inhibitors, such as ibrutinib, target Bruton’s tyrosine kinase in immune cells, including myeloid cells, modulating their interaction with T cells to enhance anti-tumor responses. A study demonstrated ibrutinib’s modulation of myeloid and T cells in metastatic PDAC, supporting its role in immune cell crosstalk (93). However, the phase III RESOLVE trial combining ibrutinib with frontline chemotherapy failed to improve survival outcomes in patients with metastatic PDAC (112). While BTK inhibition shows mechanistic promise, its clinical translation necessitates patient stratification.

### Chemokine receptor inhibitors: CXCR2 and CXCR4

In addition to the agents discussed, CXCR2 and CXCR4 inhibitors are gaining attention for their potential to modulate the PDAC TME. CXCR2 inhibitors, such as AZD5069, target neutrophil and MDSC recruitment driven by ligands like CXCL1 and CXCL5, which are upregulated in PDAC and correlate with poor outcomes (94). A phase II clinical trial (NCT02583477) is evaluating AZD5069 in combination with standard therapies in PDAC, building on preclinical evidence that CXCR2 blockade enhances T cell infiltration and chemotherapy efficacy (95). CXCR4 inhibitors (e.g., plerixafor, motixafortide) disrupt the CXCR4–CXCL12 axis, inhibiting tumor cell homing, metastasis, and immunosuppression, with clinical trials (NCT02826486, NCT03168139) showing improved T cell responses and synergy with anti-PD-1/PD-L1 therapies in metastatic PDAC (96, 113, 114). Future research on CXCR2 and CXCR4 inhibitors in PDAC should prioritize combination therapies with checkpoint inhibitors or chemotherapy, leveraging high-resolution spatial transcriptomics to map neutrophil and macrophage interactions for precise targeting. Focusing on metastatic PDAC, particularly liver metastases, will address critical needs by disrupting tumor homing and immunosuppression.

While myeloid-targeted therapeutic strategies hold considerable potential in PDAC, several scientific and clinical challenges warrant careful consideration. First, the therapeutic window of these approaches may be constrained by on-target toxicities, as exemplified by pulmonary complications observed with CCR2 inhibitor administration. Second, the heterogeneous distribution of myeloid cell subsets across patients introduces variability in treatment responsiveness, necessitating deeper biological insights. Furthermore, our current understanding of dynamic immune-stromal crosstalk remains incomplete due to technical limitations in resolving spatial cellular interactions. To address these barriers, the field is progressively exploring multimodal solutions: combining myeloid modulators with conventional chemo-radiotherapy regimens could amplify therapeutic synergy; advancing nanoparticle-based delivery systems might enhance target specificity while reducing off-tissue effects; and implementing single-cell spatial transcriptomic platforms could decode microenvironmental architecture with unprecedented resolution. Looking forward, the evolution of PDAC immunotherapy may depend on intelligently designed combination strategies that simultaneously engage multiple immunosuppressive pathways, thereby reshaping the tumor ecosystem through coordinated mechanistic interventions.

## Conclusions

To provide a comprehensive visual summary of the immunosuppressive landscape in PDAC progression, we propose schematic representations of the TME at three critical stages: primary tumor, pre-metastatic niche, and metastatic site (**Figure 1**). These schematics illustrate key cellular components (e.g., TAMs, MDSCs, neutrophils, Tregs) and molecular interactions (e.g., GM-CSF, TGF-β, CXCR2 signaling) that dominate each stage. For instance, the primary tumor schematic highlights the desmoplastic stroma and myeloid cell-driven immunosuppression, while the pre-metastatic niche emphasizes liver-specific adaptations such as Kupffer cell reprogramming via tumor-derived exosomes. The metastatic TME schematic depicts the coexistence of immunosuppressive (SPP1^+^ TAMs) and inflammatory (FCN1^+^ TAMs) macrophage subsets within spatially distinct tumor regions. These figures integrate findings from recent scRNA-seq and spatial transcriptomic studies (38, 46, 66), offering a roadmap for targeting stage-specific myeloid vulnerabilities.

In conclusion, the immunosuppressive environment of PDAC presents significant challenges for effective cancer treatment. This review highlights several key findings and future directions in the field of PDAC research and therapy. PDAC is characterized by a highly immunosuppressive TME, which includes diverse myeloid cells such as TAMs and MDSCs. These cells suppress anti-tumor immunity and promote tumor progression. Advancements in scRNA-seq and spatial transcriptomics have revealed the molecular complexity and heterogeneity of PDAC, showing dynamic changes in the TME. Studies have identified distinct immune cell populations and their interactions, providing insights into immune suppression mechanisms and potential therapeutic targets. Metastatic sites, particularly the liver, undergo microenvironmental changes that support metastatic growth even before metastasis is clinically evident. These pre-metastatic niches are characterized by the recruitment of immunosuppressive cells, such as MDSCs, macrophages, and Tregs. Targeting myeloid cells in PDAC has shown promise in enhancing anti-tumor immunity. CD40 agonists, CSF-1R inhibitors, and other novel therapeutic approaches have demonstrated potential in reprogramming macrophages and improving immunotherapy efficacy. These strategies aim to shift TAMs from an M2 to an M1 phenotype, enhance CTL infiltration, and prevent tumor growth. Future research should focus on higher-resolution spatial transcriptomics to capture the complexities of cellular interactions within the TME. Exploring combination treatments that target multiple immunosuppressive pathways simultaneously could enhance immunotherapy efficacy. Developing tailored therapies that focus on specific immunosuppressive pathways, such as the Fas–FasL pathway or the efferocytosis gene signature, could help overcome immune resistance in PDAC. By addressing these future directions, researchers can expand our insight into PDAC biology and develop more successful therapeutic strategies to overcome the immunosuppressive barriers of the TME, ultimately improving patient outcomes.
